# Mitochondria and Epigenetic Regulation: Bidirectional Crosstalk and Emerging Mitochondria-Targeted Degron Tools

**DOI:** 10.3390/cells15020095

**Published:** 2026-01-06

**Authors:** Yingwei Xu, Xiaokun Jian, Lei Shi, Lisa S. Shock, Lanming Chen, Louise T. Chow, Hengbin Wang

**Affiliations:** 1 Department of Internal Medicine, Division of Hematology, Oncology and Palliative Care, Massey Comprehensive Cancer Center, Virginia Commonwealth University, Richmond, VA 23298, USA; yingweixu2023@gmail.com (Y.X.); jxk8097@gmail.com (X.J.); doudou246@163.com (L.S.); lisa.shock@vcuhealth.org (L.S.S.); 2 College of Food Science and Technology, Shanghai Ocean University, Shanghai 201306, China; lmchen@shou.edu.cn; 3 School of Basic Medical Sciences, Xinxiang Medical University, Xinxiang 453003, China; 4 Department of Microbiology and Immunology, School of Medicine, Virginia Commonwealth University, Richmond, VA 23284, USA; 5 Department of Biochemistry and Molecular Genetics, University of Alabama at Birmingham, Birmingham, AL 35294, USA; ltchow@uab.edu

**Keywords:** epigenetics, mitochondria, gene expression, metabolism

## Abstract

**Highlights:**

**What are the main findings?**

- Mitochondrial metabolites control nuclear epigenetic marks, and nuclear factors also act on mitochondria.
- The review summarizes this two-way signaling and presents mitochondria-targeted degron tools.

**What are the implications of the main findings?**

- It links cellular metabolism directly to gene regulation.
- It offers tools to test and fine-tune mito-nuclear communication.

**Abstract:**

Mitochondria not only generate ATP and metabolites essential for nuclear and cytoplasmic processes but also actively shape nuclear epigenetic regulation. Conversely, the nucleus encodes most of the proteins required for mitochondrial functions, and intriguingly, certain nuclear-encoded epigenetic factors—such as DNA and histone modifiers—also localize to mitochondria, where they modulate mitochondria genome stability, gene expression, metabolic flux, and organelle integrity. This reciprocal interplay defines mitochondria as both a source and a target of epigenetic regulation, integrating energy metabolism with gene expression and cellular homeostasis. This review highlights emerging mechanisms that link mitochondrial metabolism to chromatin remodeling, DNA and histone modifications, and transcriptional control, as well as how nuclear epigenetic enzymes translocate into mitochondria and regulates their functions. We also briefly introduce recent methodological advances that enable spatially selective depletion of mitochondrial proteins, offering new tools to dissect this bidirectional communication. Together, these insights underscore mitochondria’s central role as an energetic and epigenetic hub coordinating nuclear function, development, and disease.

## 1. Introduction

**Figure 1:**
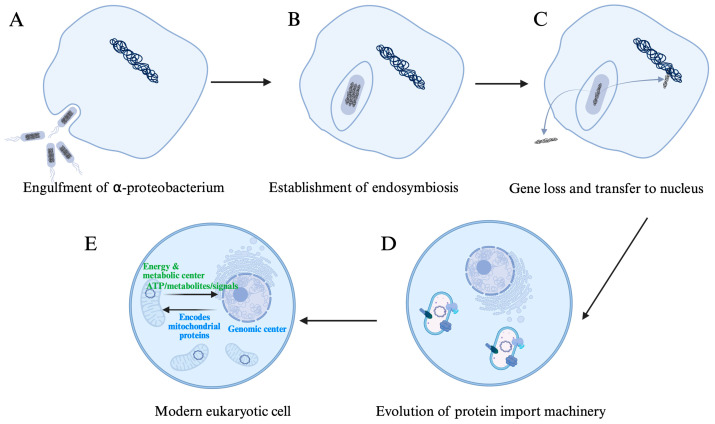
A hypothetical origin of a modern eukaryotic cell containing a nucleus and multiple copies of mitochondria. (**A**,**B**). establishing endosymbiosis. Via cell membrane invagination to enclose a small α-proteobacterium, an ancestral large proto-eukaryotic cell engulfed its prey. The prey persisted within the host cytoplasm without being digested, representing the establishment of an endosymbiotic relationship. (**C**). Gene loss and transfer. The endosymbiont, i.e., the future mitochondrion underwent genome reduction, either by transferring its genes to the host nuclear genome or through loss to the external environment. (**D**). Evolution of the cellular, nuclear, and mitochondrial membranes. The cell membrane transitions from general-purpose functions to a plasma membrane consisting of a phospholipid bilayer, specialized in material exchange and signal reception. The nucleus, defined by double-layered nuclear envelope, which enclosed the host chromosome and harbored nuclear pore complexes, stabilized into a functional compartment, controlling import and export of substrates and proteins. Mitochondria retained their double-membrane bacterial relic. Complex protein translocation systems emerged in the mitochondrial membranes, enabling accurate import and insertion of nuclear-encoded mitochondrial proteins. The inner membrane of the mitochondrion developed cristae and specialized in energy and substrate production for export into the cytoplasm and nucleus. (**E**). Evolution of the cellular organelles. Within the nucleus, a linear chromosomal DNA in association with histones and a nucleolus for rRNA synthesis, served as the genetic and metabolic control center of the cell. The endoplasmic reticulum and the Golgi apparatus became more developed, centralizing protein synthesis, processing, and sorting. Mitochondrion evolved to maintain a high copy number to increase its functional output. The modern, fully evolved eukaryotic cell is thus established. Created in BioRender. Xu, Y. (2026) https://BioRender.com/tnvanfb (accessed on 27 November 2025).

About 1.8–2 billion years ago, an ancient archaeal host cell—likely related to the Asgard lineage—engulfed a free-living α-proteobacterial ancestor and, instead of digesting it, established a stable endosymbiotic relationship (Figure 1A,B). Over time, the engulfed bacterium gradually relinquished many autonomous functions and became fully integrated into the host cell, ultimately evolving into the modern mitochondrion [1,2,3]. During this endosymbiotic transition, many genes from the bacterial endosymbiont were either lost or transferred to the host genome—a process which streamlines organelle replication and ensures stable inheritance through cytoplasm (Figure 1C). Phylogenomic analyses show that this redistribution centralizes genetic control within the nucleus and improves metabolic coordination [4,5,6]. This evolutionary transition enables the host cell to harness aerobic respiration and efficient ATP production, and is widely viewed as a defining milestone in the origin of eukaryotic complexity [7,8,9].

In human cells, the mitochondrial genome (mtDNA) has been reduced to a circular DNA molecule of ~16.6 kb (NC_012920), encoding only 13 proteins for oxidative phosphorylation, 22 transfer RNAs (tRNAs), and 2 ribosomal RNAs (rRNAs) [10,11,12]. This organization supports local synthesis and rapid assembly of hydrophobic core subunits of respiratory complexes into the inner mitochondrial membrane [10]. Despite its small gene content, mtDNA is maintained at high copy number per cell [13,14,15]. Importantly, this copy number, together with mtDNA integrity, is strongly influenced by mitochondrial fusion–fission dynamics. Consistent with this, loss of mitofusin-dependent outer-membrane fusion impairs mtDNA replication and leads to a marked decline in mtDNA content in proliferating cells and during postnatal cardiac development, indicating that fusion is required for faithful replication and distribution of the mitochondrial genome [16]. Clinically, this principle is illustrated by a rare human MFN2 R400Q variant that perturbs mitofusin function and Parkin-mediated mitophagy, causing progressive cardiomyopathy in knock-in mice and being enriched in human cardiomyopathy cohorts [17]. In contrast, the vast majority of mitochondrial proteins, numbering in thousands, are nuclear-encoded [11], synthesized in the cytosol, and imported into the organelle via translocases of the outer and inner membranes (TOM/TIM complexes) [12] (Figure 1D). This tight interdependence has forged a coevolutionary relationship between the nuclear and mitochondrial genomes: the nucleus provides the biosynthetic and regulatory machinery for mitochondrial function, while mitochondria, in turn, supply the nucleus with ATP [18], key metabolic intermediates such as acetyl-CoA and α-ketoglutarate [19,20,21], reactive oxygen species (ROS) that act as signaling molecules [22], and additional intermediates that modulate chromatin dynamics and nuclear function [23] (Figure 1E).

Contemporary evolutionary analyses further suggest that this mitochondrion–nucleus symbiosis is a driving force behind eukaryotic evolution. The endosymbiotic partnership imposes selective pressures that promote genomic integration, signaling interdependence, and metabolic specialization—ultimately catalyzing the rise of cellular complexity [5,9,24]. This enduring intergenomic dialogue continues to shape cell physiology, development, and disease, underscoring mitochondria’s central role as both an energetic and epigenetic integrator.

## 2. Mitochondria as a Producer of Energy and Signaling Metabolites

**Figure 2:**
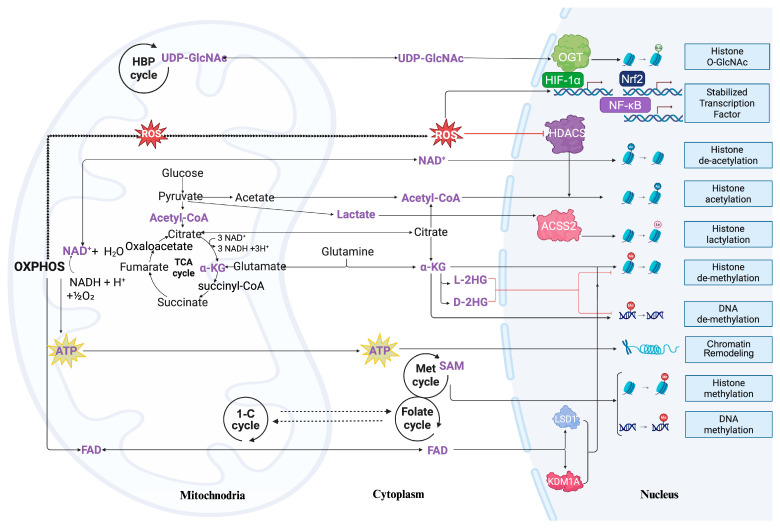
Crosstalk between mitochondrial metabolism and nuclear epigenetic regulation. Mitochondria generate ATP through oxidative phosphorylation (OXPHOS) and supply key metabolites, including NAD^+^, acetyl-CoA, and α-ketoglutarate (α-KG), via the tricarboxylic acid (TCA) cycle. In parallel, one-carbon (1-C) metabolism and the methionine/folate cycles produce S-adenosylmethionine (SAM). These metabolites are exported into the cytoplasm and nucleus, where they act as essential cofactors for chromatin-modifying enzymes. ATP contributes to chromatin remodeling; NAD^+^ regulates sirtuin-mediated deacetylation; acetyl-CoA fuels histone acetylation; α-KG serves as a cofactor for Jumonji C (JmjC)-domain histone demethylases and TET DNA demethylases; and SAM donates methyl groups for histone and DNA methylation. The scheme also includes a glycolytic pyruvate–lactate branch. A glycolytic side branch converts pyruvate to lactate when mitochondrial pyruvate oxidation is limited; in the nucleus, ACSS2 uses lactate to generate lactyl-CoA that supports histone lactylation. Within mitochondria, ROS is generated, while oxidative phosphorylation provides FAD as a redox cofactor. The hexosamine biosynthetic pathway (HBP) produces UDP-GlcNAc. These metabolites are transferred to the nucleus through distinct signaling routes: mtROS enter as redox signals, while UDP-GlcNAc and FAD diffuse or are transported. In the nucleus, mtROS inhibit histone methylation by oxidatively modifying the SET1/MLL complex and stabilizing transcription factors such as HIF1α, Nrf2, and NF-κB. UDP-GlcNAc serves as a substrate for O-GlcNAc transferase (OGT), promoting histone O-GlcNAcylation. FAD functions as an essential cofactor for the demethylases LSD1/KDM1A, which catalyze histone demethylation and suppress transcription. Created in BioRender. Xu, Y. (2026) https://BioRender.com/tnvanfb (accessed on 28 December 2025).

Mitochondria are classically recognized as the cell’s powerhouse, generating ATP to drive essential processes such as transcription, replication, and DNA damage repair. In addition, mitochondrial metabolism produces key intermediates—most notably citrate and acetyl-CoA—that fuel major biosynthetic pathways, including fatty acid, cholesterol, and steroid synthesis. Importantly, many of these same metabolites also act as signaling cofactors that directly influence chromatin structure and nuclear gene expression. The following section explores how mitochondria-derived ATP and metabolites mechanistically couple cellular metabolic state to epigenetic regulation (Figure 2).

### 2.1. Adenosine Triphosphate (ATP)

ATP, the universal energy currency of the cell, is generated predominantly through mitochondrial oxidative phosphorylation (OXPHOS), with additional contributions from glycolysis [25,26,27]. Continuous ATP production is essential to meet cellular energetic and biosynthetic demands, sustaining both catabolic and anabolic processes.

Within the nucleus, ATP supplies energy for nearly all processes that safeguard genome integrity and regulate gene expression. During DNA replication, ATP binding and hydrolysis by helicases such as the MCM complex and by clamp-loader ATPases (RFC, CTF18-RFC, RAD17-RFC, and ATAD5-RFC) drive origin licensing, helicase activation, and proliferating cell nuclear antigen (PCNA) loading [28,29,30,31]. During transcription, ATP provides the requisite energy for the assembly, initiation, and elongation phases of RNA polymerases, processes essential for maintaining transcriptional fidelity and regulating transcriptional pausing [32,33]. Chromatin remodeling complexes, including SWI/SNF, ISWI, INO80, and CHD families, convert ATP hydrolysis into mechanical force to reposition or evict nucleosomes, thereby modulating DNA accessibility for transcription, replication, and repair [34,35,36]. Similarly, DNA repair mechanisms, including homologous recombination and non-homologous end joining, rely on ATPases like CSB and clamp-loader complexes to coordinate lesion detection, processing, and rejoining [37,38,39]. Beyond DNA metabolism, ATP hydrolysis also energizes RNA splicing through DEAD-/DExH-box RNA helicases and supports nucleocytoplasmic transport via the karyopherin/Ran GTP-ATP regulatory cycles, ensuring the directional trafficking of macromolecules across the nuclear envelope [40,41,42].

Emerging evidence indicates that mitochondrial ATP fluctuations directly influence nuclear chromatin architecture and transcriptional programs. ATP depletion reduces chromatin mobility and disrupts nuclear organization [43,44], whereas mitochondria-derived nuclear ATP surges under mechanical confinement preserve chromatin accessibility and facilitate DNA repair [44,45]. In tissues with high energy demand—such as the brain, heart, and muscles—ATP insufficiency rapidly compromises neuronal signaling, cardiac contractility, and energy metabolism, culminating in cellular dysfunction and apoptosis [46,47,48,49,50].

Moreover, ATP-dependent chromatin remodelers can act as direct metabolic sensors that couple cellular energy status to gene regulation and genome maintenance. Under stress conditions, remodelers such as INO80 and SWI/SNF undergo structural and functional changes that facilitate nucleosome repositioning and transcriptional reprogramming to preserve genome stability [35,36,51]. Collectively, by linking its synthesis to virtually all vital nuclear processes, ATP functions as the central energetic hub connecting cellular metabolism to chromatin dynamics and genome regulation.

### 2.2. Acetyl-CoA

Within mitochondria, acetyl-CoA is synthesized through multiple converging metabolic pathways. The primary origin is the oxidative decarboxylation of pyruvate facilitated by the pyruvate dehydrogenase complex [52,53]. Additional sources include fatty acid β-oxidation, ketone body metabolism, and the catabolism of ketogenic and glucogenic amino acids, such as leucine (ketogenic), isoleucine (both ketogenic and glucogenic), and valine (glucogenic) [54,55,56]. Acetate can also be transformed into acetyl-CoA via mitochondrial acyl-CoA synthetase short-chain family member 1, thereby ensuring adaptability in response to fluctuating nutrient conditions [57,58,59,60]. Together, these pathways guarantee a continual supply of acetyl-CoA, establishing it as a metabolic node that integrates nutrient availability, energy production, and biosynthetic capacity—supplying substrates for fatty acid, cholesterol, and steroid synthesis—while simultaneously providing the acetyl donor that links mitochondrial metabolism to nuclear epigenetic regulation [57,61].

Because acetyl-CoA cannot cross the mitochondrial inner membrane directly, cells use citrate as a carbon shuttle. Citrate is exported to the cytosol via the citrate carrier SLC25A1 and cleaved by ATP-citrate lyase (ACLY) to regenerate cytosolic and nuclear acetyl-CoA [62,63,64]. This extramitochondrial acetyl-CoA pool serves as the acetyl donor for protein acetylation reactions, notably histone acetylation, a fundamental epigenetic modification that modulates chromatin conformation and transcriptional activity [65]. Histone acetyltransferases (HATs), including CBP/p300, GCN5, and MOF, transfer acetyl groups from acetyl-CoA to lysine residues on histone tails, neutralizing their positive charge and weakening histone-DNA interactions. Consequently, a more relaxed chromatin structure facilitates transcription factor recruitment and RNA polymerase II access, leading to transcriptional activation [66,67].

Under nutrient-rich conditions, elevated acetyl-CoA levels enhance histone acetylation, activating transcriptional programs that promote cell growth, biosynthesis, and proliferation [57,68]. Conversely, during nutrient deprivation or mitochondrial dysfunction, acetyl-CoA is preferentially oxidized in the tricarboxylic acid (TCA) cycle to sustain ATP synthesis, thereby reducing the nuclear acetyl-CoA pool. This depletion leads to hypoacetylation of histones, chromatin compaction, and transcriptional repression of anabolic and growth-associated genes [19,66,69].

Recent evidence emphasizes that mitochondrial acetyl-CoA flux dynamically regulates histone acetylation in a locus-specific manner. Mitochondrial perturbations or loss of mtDNA alter citrate export and acetyl-CoA availability, producing selective changes in histone acetylation and gene expression [67,70]. Intriguingly, histone acetylation itself may serve as a metabolic buffer, storing acetate that can be mobilized back into acetyl-CoA under stress, suggesting a bidirectional relationship between histone modification and metabolism [69]. Furthermore, emerging research links tissue-specific acetyl-CoA metabolism to epigenetic and immune regulation. In macrophages, mitochondrial fatty acid oxidation via ACAT1 (Acetyl-CoA Acetyltransferase 1) generates acetyl-CoA that drives histone acetylation and transcription of type I interferon–responsive genes, demonstrating a direct mechanistic link between mitochondrial metabolism and nuclear immune signaling [62,71]. Together, these studies establish acetyl-CoA as both a metabolic substrate and a signaling molecule that couples mitochondrial energy metabolism to nuclear chromatin remodeling and transcriptional control.

### 2.3. Histone Lactylation

Histone lactylation is a recently identified post-translational modification that links glycolytic metabolism to chromatin remodeling and gene regulation. Zhang et al. (2019) first reported lysine lactylation (Kla) on histone tails, where lactate-derived lactyl groups, catalyzed by the histone acetyltransferase p300 using lactyl-CoA, modify lysine residues such as H3K18la and directly stimulate transcription of homeostatic and wound-healing genes during late-phase macrophage polarization [72].

Subsequent work has greatly expanded the biological scope of Kla. In cancer, histone lactylation is increasingly recognized as a key mediator that couples tumor-associated lactate accumulation (a consequence of aerobic glycolysis and altered mitochondrial metabolism) to oncogenic transcription programs and immune modulation in the tumor microenvironment, emphasizing lactate as a signaling metabolite rather than a mere metabolic by-product [73,74]. In innate immunity, Ziogas et al. [75] showed that lactate-driven H3K18la persists in monocytes for at least 90 days after BCG vaccination, serving as a long-lived epigenetic mark of trained innate immune memory. Lactate-induced H3K18la also promotes macrophage M1 polarization and vascular inflammation in models of abdominal aortic aneurysm, illustrating how Kla connects local metabolic changes to chronic inflammatory disease [76].

Lactylation is likewise observed in the nervous system, where neuronal excitation and social stress elevate brain lactate levels and increase histone H1 lactylation and other Kla marks in cortical neurons. These changes correlate with altered social and anxiety-like behaviors, suggesting that neuronal lactate–Kla signaling contributes to activity-dependent chromatin remodeling and neural plasticity [77,78].

At the metabolic level, the pool of lactate available for histone lactylation is governed by the balance between glycolytic pyruvate production and mitochondrial pyruvate oxidation. When mitochondrial respiration is constrained, more pyruvate is reduced to lactate by lactate dehydrogenase, thereby increasing substrate availability for Kla. Conversely, efficient mitochondrial oxidation of pyruvate limits lactate accumulation and may restrain Kla [79]. Recent work further shows that the acyl-CoA synthetase ACSS2 can act as a lactyl-CoA synthetase in the nucleus and cooperate with KAT family lysine acyltransferases to install Kla on histone H3 and H4, establishing a direct enzymatic route from lactate to histone lactylation [80]. Collectively, histone lactylation exemplifies how changes in mitochondrial and glycolytic metabolism are decoded at the chromatin level to regulate gene expression across immunity, neurobiology, and tumorigenesis.

### 2.4. Acyl-CoAs

In addition to acetyl-CoA, a variety of acyl-CoA derivatives—including propionyl-CoA, butyryl-CoA, crotonyl-CoA, succinyl-CoA, and malonyl-CoA—also serve as donors for diverse histone acylations, thereby expanding the epigenetic regulatory landscape. These acyl-CoAs originate from the oxidation of fatty acids with varying chain lengths, amino acid catabolism, and intermediates of the TCA cycle, thereby linking cellular metabolism with chromatin state [81,82]. For instance, propionyl-CoA is generated from the breakdown of odd-chain fatty acids and specific amino acids (valine, isoleucine, methionine, and threonine) and is subsequently converted to succinyl-CoA for entry into the TCA cycle [83]. Butyryl-CoA and crotonyl-CoA serve as intermediates in short-chain fatty acid metabolism and function as acyl donors for histone butyrylation and crotonylation, respectively. These two modifications are enriched at active promoters and correlate with transcriptional activation [84,85,86,87,88]. Succinyl-CoA (an intermediate in the TCA cycle) and malonyl-CoA (a key intermediate in fatty acid synthesis) can also serve as acylation donors. They participate in protein post-translational modifications such as lysine succinylation and malonylation, modifications that can alter enzyme activity, stability, and localization [89,90,91,92,93].

These non-acetyl acylations provide a direct mechanistic link between mitochondrial metabolism and chromatin dynamics, enabling cells to sense metabolic flux and translate it into epigenetic changes [81,90,94]. The relative abundance of these acyl-CoA species is highly sensitive to nutrient availability, oxygen status, and mitochondrial function, thereby permitting fine-tuned regulation of gene expression in response to stimuli [81,94,95]. Thus, these acyl-CoAs are not just metabolic intermediates but active modulators of transcription, DNA repair, and chromatin architecture across developmental, pathological, and aging processes [96].

### 2.5. NADH/NAD^+^

Within mitochondria, NAD^+^ serves as a central redox coenzyme, cycling between its oxidized (NAD^+^) and reduced (NADH) forms. Cytoplasmic NAD^+^ supports glycolysis, while mitochondrial NAD^+^ supports the TCA cycle, fatty acid β-oxidation, and the electron transport chain (ETC) [97]. In this capacity, NADH supplies reducing equivalents that drive the oxidative phosphorylation and ATP synthesis [98]. Beyond its conventional redox activity, NAD^+^ is also consumed as a substrate for nuclear and cytoplasmic enzymes, notably sirtuins and poly (ADP-ribose) polymerases (PARPs), thereby linking metabolic status to chromatin dynamics and DNA repair mechanisms [99,100].

Histone deacetylation represents a fundamental axis of metabolic-epigenetic coupling. Class I and II histone deacetylases (HDACs) remove acetyl groups independent of NAD^+^, inducing chromatin condensation and transcriptional repression [101,102]. In contrast, Class III HDACs, known as sirtuins, uniquely require NAD^+^ as a cofactor for their deacetylase activity. Fluctuations in the NAD^+^/NADH ratio consequently modulate sirtuin activity and downstream epigenetic outcomes [102,103,104,105,106,107]. For example, nuclear sirtuins (SIRT1 and SIRT6) deacetylate histone marks such as H3K9Ac and H3K56Ac, enforcing chromatin compaction, repressing transcription, and promoting DNA repair [108]. Mitochondrial Sirtuin 3 (SIRT3), by contrast, targets respiratory chain proteins and metabolic enzymes, enhancing mitochondrial oxidative metabolism and antioxidant defense [109]. Together, sirtuins serve as metabolic–epigenetic integrators, translating NAD^+^ availability into coordinated nuclear and mitochondrial responses [110,111,112].

NAD^+^ likewise regulates PARP-1, which binds to nucleosomes under basal conditions and helps maintain chromatin compaction [99,113]. When NAD^+^ is abundant, it serves as the essential substrate for PARylation. DNA damage/chromatin signals trigger PARP-1 auto-PARylation, which loosens chromatin and enhances its accessibility [114,115]. Conversely, when NAD^+^ decline, such as during aging, metabolic disorders, or mitochondrial dysfunction, sirtuin activity (which normally promotes chromatin compaction via deacetylation) is compromised. Simultaneously, excessive PARP-1 activation under chronic stress can over-consume NAD^+^, further depleting the NAD^+^ pool and disrupting chromatin homeostasis. The failure of sirtuin-mediated deacetylation leads to histone hyperacetylation and aberrant chromatin relaxation, resulting in transcriptional dysregulation and activation of pro-inflammatory gene programs. In this way, NAD^+^ homeostasis serves as a pivotal connection between bioenergetic processes and nuclear epigenetic regulation, impacting cell fate decisions [99,116].

### 2.6. S-Adenosylmethionine (SAM)

The folate and methionine cycles intersect within the cytosol to generate SAM, the universal methyl donor essential for cellular DNA, RNA, and histone methylation [117,118]. SAM is synthesized from methionine via methionine adenylyltransferase (MAT) [119,120,121]. The methylation process yields S-adenosylhomocysteine (SAH), which is hydrolyzed to homocysteine and subsequently remethylated to methionine using one-carbon units supplied by mitochondrial folate metabolism. Thus, mitochondrial one-carbon metabolism, driven by serine and glycine catabolism, provides formate and various intermediates vital for cytosolic SAM synthesis [122,123].

SAM donates methyl groups to DNA methyltransferases (DNMTs), RNA methyltransferases (RNMTs), and histone methyltransferases (HMTs), while the byproduct SAH functions as a potent feedback inhibitor of these enzymes. Thus, the intracellular SAM/SAH ratio is a sensitive determinant of methylation potential across the cytosol, nucleus, and mitochondria [124,125]. Elevated SAM levels or high SAM/SAH ratio promote methylation of histones (e.g., H3K4me3, H3K36me3), RNA (m^6^A), and DNA (5mC), supporting transcriptional fidelity and chromatin stability [118,126,127]. In contrast, mitochondria dysfunction or limitations in one-carbon flux can decrease SAM levels and reduce SAM/SAH ratio, suppress methylation reactions, and lead to chromatin and transcription dysregulation [121,128,129,130]. In disease and aging, alterations in one-carbon metabolism and deficiencies in SAM are associated with impaired histone methylation, DNA hypomethylation, and transcriptional noise, contributing to oncogenesis, neurodegeneration, and immune dysfunction [130,131,132]. Nutrient-dependent regulation of one-carbon metabolism—involving intake of methionine, folate, and serine—has also been shown to restore methylation equilibrium and epigenetic stability, suggesting potential therapeutic interventions for metabolic and epigenetic disorders [121].

Emerging research emphasizes the significance of mitochondrial import of SAM as a pivotal step in regulating mitochondrial gene expression and protein methylation. SAM transporters located within the mitochondrial membrane facilitate localized methylation of mitochondrial tRNAs and proteins, directly linking cytosolic methyl donor availability to mitochondrial translation and respiration [133,134]. This bidirectional metabolic exchange allows mitochondria not only to function as energy producers but also to serve as regulators of epigenetic states through one-carbon metabolism and SAM flux. Overall, SAM functions as an integral metabolic–epigenetic nexus, translating mitochondrial one-carbon flux into chromatin methylation dynamics that govern gene expression, development, and cellular plasticity.

### 2.7. α-Ketoglutarate (α-KG)

α-KG is a pivotal TCA intermediate produced through isocitrate oxidative decarboxylation by isocitrate dehydrogenases (IDH2 and IDH3) or by glutamate deamination catalyzed by glutamate dehydrogenase (GLUD1). Cytosolic IDH1 also generates α-KG outside mitochondria, maintaining cross-compartmental redox and metabolic flux [135,136]. Beyond its metabolic role, α-KG serves as an essential cofactor for a wide family of α-KG/Fe(II)-dependent dioxygenases, including Jumonji C (JmjC) domain-containing histone demethylases (JMJDs) and ten-eleven translocation (TET) DNA hydroxylases [137]. These enzymes mediate oxidative demethylation of methylated lysine residues (e.g., H3K4me3, H3K27me3) and convert 5-methylcytosine (5mC) into 5-hydroxymethylcytosine (5hmC), 5-formylcytosine (5fC), and 5-carboxylcytosine (5caC), key intermediates of DNA demethylation [138,139]. The enzymatic activity of JMJDs and TETs is tightly regulated by the intracellular balance of α-KG and structurally similar TCA intermediates, notably succinate, fumarate, and the oncometabolite 2-hydroxyglutarate (2-HG), which acts as a competitive inhibitor. This biochemical antagonism establishes a direct mitochondria-epigenetic link between mitochondrial activity and chromatin dynamics [137,139,140,141].

In cancer, mutations in IDH1/2 confer neomorphic activity that converts α-KG into 2-HG, leading to inhibition of TETs and JmjC demethylases, global hypermethylation, and transcriptional repression [139,142]. In pluripotent stem cells, α-KG sustains self-renewal and promotes reprogramming by stimulating TET1/2-dependent DNA demethylation, facilitating open chromatin configurations and transcriptional plasticity [143,144]. In the immune system, macrophages exhibit metabolic polarization governed by α-KG: a high α-KG/succinate ratio drives M2-like anti-inflammatory programs via Jmjd3-mediated histone demethylation, linking metabolic state to epigenetic control of inflammation [145,146].

In neurons, α-KG-dependent TET3 activity regulates hydroxymethylation dynamics essential for synaptic plasticity and neural differentiation [147]. Within the cardiovascular system, decreased α-KG availability during hypoxia or metabolic stress limits TET-mediated DNA demethylation, leading to vascular remodeling and fibrosis [148]. During aging, mitochondrial dysfunction reduces α-KG synthesis and transport, attenuates TET/JmjC activity, and causing epigenetic drift and transcriptional instability [149,150]. Mechanistically, mitochondrial glutamine oxidation and α-KG import/export across the mitochondrial membrane constitute major regulatory levers that coordinate nuclear demethylation activity, establishing α-KG as a metabolic rheostat aligning energy metabolism with epigenetic fidelity [139,151].

Collectively, α-KG functions as a central metabolic-epigenetic mediator, synchronizing mitochondrial energetics with nuclear chromatin regulation. Alternations in α-KG metabolism—whether through IDH mutations, hypoxia, or aging—disrupt this metabolic-epigenetics axis, contributing to oncogenesis, neurodegeneration, and age-associated dysfunction.

### 2.8. Flavin Adenine Dinucleotide (FAD)

FAD is an essential redox cofactor that plays a dual role in mitochondrial energy metabolism and epigenetic regulation. Synthesized from riboflavin (vitamin B2) through the sequential actions of riboflavin kinase and FAD synthetase, FAD acts as a tightly bound flavin cofactor for various mitochondrial enzymes, including succinate dehydrogenase (complex II) in ETC and acyl-CoA dehydrogenases in fatty acid β-oxidation. In these reactions, FAD accepts electrons from metabolic substrates (e.g., succinate → fumarate) and transfers them to coenzyme Q, supporting oxidative phosphorylation and ATP synthesis [152].

Beyond energy metabolism, FAD is indispensable for the activity of flavin-dependent amine oxidase histone demethylases, most notably lysine-specific demethylase 1 (LSD1/KDM1A). LSD1 catalyzes the FAD-dependent oxidative demethylation of mono- and di-methylated lysine residues on histone H3, such as H3K4me1/2 and H3K9me1/2, contingent upon its associated cofactors and chromatin context and modulating chromatin compaction and gene expression [153,154]. This FAD-driven reaction mechanistically links mitochondrial redox homeostasis to chromatin remodeling and transcriptional control [155,156].

The LSD1/FAD axis influences key transcriptional programs governing metabolism, differentiation, and cancer. For instance, FAD-dependent LSD1 activity represses thermogenic gene expression in brown adipose tissue, promoting energy expenditure [153], while in hepatocellular carcinoma, LSD1 coordinates glycolytic and mitochondrial metabolism, supporting tumor growth [157].

Fluctuations in mitochondria FAD availability profoundly affect LSD1-mediated chromatin dynamics. Reduced FAD levels—whether from riboflavin deficiency or mitochondrial dysfunction—lead to diminished LSD1 activity, resulting in histone hypermethylation and transcriptional dysfunction [158,159]. Conversely, restoration of mitochondrial FAD pools enhances LSD1-mediated demethylation and promotes metabolic flexibility, reinforcing the metabolic-epigenetic link [160].

FAD-dependent LSD1 also demethylates non-histone substrates, including RACK1, thereby stabilizing hypoxia-inducible factor (HIF1α) and fine-tuning oxygen-sensing pathways [161]. This interaction exemplifies how FAD redox dynamics directly influence both nuclear gene regulation and cellular adaptation to hypoxia. Recent findings indicate that FAD-dependent LSD1 activity declines with age due to impaired riboflavin uptake and mitochondrial dysfunction, contributing to chromatin relaxation, senescence, and transcriptional noise [160,162]. Dysregulation of this axis has been linked to neurodegenerative diseases and cancer, where disrupted FAD metabolism alters histone methylation landscapes and impairs energy balance [163].

Collectively, these findings position FAD as a metabolic–epigenetic integrator that connects mitochondrial redox homeostasis with chromatin remodeling. Through regulation of LSD1 and other FAD-dependent demethylases, mitochondrial metabolism directly influences transcriptional regulation, developmental programming, and disease states, such as cancer, neurodegeneration, and aging.

### 2.9. UDP-N-Acetylglucosamine (UDP-GlcNAc)

UDP-GlcNAc is the terminal product of the hexosamine biosynthetic pathway (HBP), a metabolic branch that integrates nutrient inputs from glucose, amino acids (particularly glutamine), fatty acids, and nucleotide metabolism. Due to this integration, UDP-GlcNAc serves as a sensitive indicator of cellular nutrient and energy status, acting as a metabolic gauge for growth and stress signaling [164,165]. The primary function of UDP-GlcNAc is to serve as the donor substrate for O-linked β-N-acetylglucosamine (O-GlcNAc) modification—a dynamic and reversible post-translational modification of nuclear, cytosolic, and mitochondrial proteins catalyzed by O-GlcNAc transferase (OGT) and removed by O-GlcNAcase (OGA) [166].

OGT utilizes UDP-GlcNAc to modify a wide range of substrates, including histones, transcription factors, and chromatin remodelers, making O-GlcNAcylation serves as a nutrient-sensitive epigenetic switch that couples metabolic flux to gene expression [167,168]. Histone residues such as H2B-S112, H3-T32, and H4-S47 have been identified as O-GlcNAcylation sites that can alter nucleosome stability, histone acetylation, and methylation patterns, thereby fine-tuning chromatin accessibility and transcriptional dynamics [165,168].

Levels of HBP flux and UDP-GlcNAc availability directly modulate OGT activity, influencing chromatin remodeling and transcriptional homeostasis. Under nutrient-rich conditions, elevated glucose and amino acid levels increase UDP-GlcNAc synthesis, leading to hyper-O-GlcNAcylation of transcriptional regulators and histone modifiers. This phenomenon has been linked to insulin resistance, tumor progression, and aging-related dysfunction [169,170,171]. Conversely, pharmacologic or genetic OGT inhibition restores chromatin accessibility and reprograms transcriptional activity, offering potential therapeutic strategies in cancer, neurodegeneration, and metabolic diseases [172,173].

Mechanistically, OGT interacts with TET2/3 and SET1/COMPASS, coordinating DNA demethylation and histone methylation at active gene promoters. This cross-regulatory mechanism integrates O-GlcNAcylation, DNA hydroxymethylation, and histone methylation, enabling dynamic transcriptional responses to metabolic flux [167,174]. Recent work extends this axis to ferroptosis, stem cell pluripotency, and redox signaling, where OGT-dependent modifications regulate cell fate, stress resistance, and mitochondria integrity [170,175]. Together, UDP-GlcNAc and OGT represent a central metabolic-epigenetic hub that translates nutrient availability into chromatin state and transcriptional programs. Through O-GlcNAcylation, cells synchronize metabolic flux, redox balance, and gene expression, maintaining adaptive homeostasis across physiological and pathological contexts.

### 2.10. Reactive Oxygen Species (ROS)

ROS—including superoxide (O_2_•^−^), hydrogen peroxide (H_2_O_2_), and hydroxyl radicals (•OH)—are both byproducts and essential regulators of aerobic metabolism [176,177,178,179]. Mitochondria represent the primary intracellular source of ROS, which is produced through electron leakage at complexes I and III of the ETC during oxidative phosphorylation [180,181,182]. While excessive ROS can cause oxidative damage to DNA, proteins, and lipids, physiological ROS act as signaling molecules that link mitochondrial activity to epigenetic regulation and chromatin remodeling [22,183,184]. This redox-epigenetic coupling ensures dynamic control of gene expression in response to metabolic and environmental cues.

Recent studies emphasize ROS as a mitochondria-to-nucleus messenger, regulating chromatin structure and epigenetic plasticity. Oxidative stress modulates Fe (II)/α-KG-dependent dioxygenases, including TET DNA demethylases and JmjC-domain histone demethylases, through oxidizing catalytic Fe (II) or depleting α-KG, resulting in localized chromatin compaction and transcriptional repression under oxidative stress [185,186]. Conversely, moderate ROS levels activate redox-sensitive transcription factors such as NRF2, NF-κB, and HIF-1α, which regulate antioxidant responses, inflammation, and metabolic reprogramming [148,187]. At the chromatin level, oxidative inhibition of HDACs enhances histone acetylation and promotes transcriptional activation of stress-responsive genes [175,186,188]. Moreover, lysyl oxidase-like 2 (LOXL2)-mediated H3K4 oxidation (H3K4ox) is linked to heterochromatin compaction and reduced accessibility, suggesting that histone oxidation represents a novel regulatory layer of chromatin [189].

ROS fine-tune the activity of sirtuins (SIRT1, SIRT3), which acts as NAD^+^-dependent redox sensors bridging mitochondrial metabolism with nuclear transcriptional programs. SIRT3 attenuates mitochondrial ROS production by deacetylating and activating antioxidant enzymes, while SIRT1 couples redox signaling to histone deacetylation and stress-responsive gene regulation [91,190]. In addition, oxidative DNA lesions such as 8-oxo-2′-deoxyguanosine (8-oxo-dG) recruit TET enzymes and DNA repair complexes, establishing a mechanistic link between oxidative damage and DNA demethylation [191].

In the context of aging, persistent oxidative stress contributes to epigenetic drift—global alterations in histone methylation (e.g., H3K4me3, H3K27me3) and DNA methylation—that reduce chromatin integrity and increase transcriptional noise [192]. In cancer, chronic oxidative stress disrupts normal redox-epigenetic homeostasis, driving tumor progression through aberrant methylation and acetylation of oncogenic pathways [184,193,194]. Dietary antioxidants and polyphenols can reverse ROS-induced epigenetic alterations, restoring chromatin organization, and suppressing carcinogenic gene expression [195]. Nonetheless, physiological ROS signaling enhance stress resilience and longevity by activating adaptive transcriptional programs and redox-protective chromatin remodeling [182].

Overall, ROS serve as dual-function molecules—deleterious at excessive levels but indispensable for redox–epigenetic homeostasis under physiological conditions. Through modulation of histone-modifying enzymes, transcription factors, and DNA repair machinery, ROS integrate mitochondrial metabolism with nuclear gene expression, forming a core axis of metabolic-epigenetic communication that governs cell fate, adaptation, and disease.

## 3. Mitochondria Localization of Nuclear Proteins

**Figure 3:**
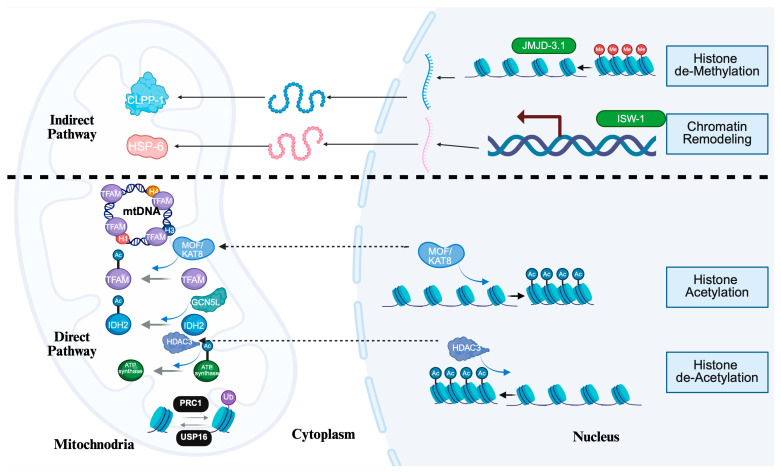
Indirect and direct epigenetic pathways regulating mitochondrial functions. **Top**: Nuclear chromatin modifications control the expression of mitochondrial proteins, thus modulate mitochondrial function indirectly. The histone demethylase JMJD-3.1 removes the repressive H3K27me3 mark to activate transcription of the mitochondrial quality control gene CLPP1, while the chromatin remodeler ISW-1 coordinates transcription of the mitochondrial chaperone gene HSP6. Once activated, these genes are transcribed into mRNA, translated in the cytoplasm, and the proteins are imported into mitochondria, thereby indirectly influencing mitochondrial activity. **Bottom**: Epigenetic regulators and histones localize in mitochondria and modulate mitochondrial function directly. Mitochondrial DNA (mtDNA) is organized into nucleoid-like structures by TFAM together with histone-like proteins. The MOF/KAT8 complex acetylates TFAM to promote mtDNA transcription. GCN5L, acetylates the TCA cycle enzyme IDH2 to regulate metabolism. HDAC3 removes acetyl groups from nuclear histones and from mitochondrial ATP synthase to prevent over-activation. In addition, mitochondria may contain histones undergoing ubiquitination and deubiquitination: the PRC1 complex ubiquitinates mitochondrial histones to influence mtDNA packaging and transcription, whereas USP16 deubiquitinates mitochondrial H2A. Together, these indirect and direct pathways highlight how epigenetic mechanisms regulate mitochondrial function at multiple levels. Created in BioRender. Xu, Y. (2026) https://BioRender.com/tnvanfb (accessed on 27 November 2025).

Traditionally, epigenetic regulators—such as DNA methyltransferases, histone-modifying enzymes, and chromatin remodelers—were viewed as strictly nuclear factors that regulate chromatin structure and transcription. However, emerging evidence over the past decade reveals that several of these nuclear enzymes exhibit dual localization in the nucleus and mitochondria, suggesting that epigenetic control extends beyond the nuclear genome [139,196]. Mitochondrial localization has now been documented for DNA methyltransferase 1 (DNMT1), ten-eleven translocation (TET) dioxygenases, and certain HDACs—notably sirtuins (SIRT3–SIRT5)—which are key regulators of mitochondrial protein acetylation and redox balance [197,198,199,200]. These proteins contribute to mtDNA methylation, transcriptional regulation of mtDNA-encoded genes, and post-translational modification of metabolic enzymes [201,202] (Figure 3).

This dual compartmentalization implies that epigenetic regulators not only shape nuclear transcriptional programs but also directly influence mitochondrial metabolism, redox homeostasis, and mtDNA organization [196,203]. These epigenetic regulators may act indirectly by regulating the expression of nuclear-encoded mitochondrial genes, or directly by modifying mitochondrial proteins and mtDNA within the organelle [204]. Together, these findings underscore a bidirectional regulatory axis in which nuclear epigenetic factors contribute to the coordination of mitochondria-nuclear communication, ensuring that cellular energy metabolism and gene expression remain synchronized in response to environmental and metabolic cues [205,206,207]. These proteins are discussed in detail below.

### 3.1. DNA Methyltransferase 1

Human mtDNA has a GC content of ~44% and pronounced strand asymmetry, with the heavy (H) strand being guanine-rich and the light (L) strand cytosine-rich [208]. Historically, mtDNA organization is viewed as nucleoid structures rather than chromatin, packaged by TFAM instead of histones, and transcribed as polycistronic transcripts from the displacement-loop (D-loop) region, which serves as the central control site for replication and transcription [209,210]. Subsequent compositional and sequencing studies revealed mtDNA’s low CpG density and asymmetric replication mechanism, where the prolonged single-stranded exposure of the H-strand contributes to its unique base composition and elevated mutation rate [211].

Initially, mtDNA was considered epigenetically inert. This perspective changed dramatically with the discovery of a mitochondrial isoform of DNA methyltransferase 1 (mtDNMT1) [212]. mtDNMT1 contains an extended N-terminal mitochondrial targeting sequence that directs it to the mitochondrial matrix, where it binds to CpG-rich regions of mtDNA—including the D-loop—and catalyzes cytosine methylation (5mC). This study also revealed that mtDNMT1 expression is transcriptionally regulated by oxidative stress–responsive transcription factors such as NRF1, PGC1α, and p53, thereby positioning mtDNA methylation as a redox-sensitive epigenetic process linking mitochondrial status to gene expression. Moreover, the detection of 5-hydroxymethylcytosine (5-hmC) in mtDNA implicates TET enzyme activity within mitochondria, suggesting an active methylation-demethylation cycle.

Over the past decades, biotechnology has expanded rapidly. High-resolution bisulfite sequencing, nanopore long-read mapping, and oxidative-bisulfite conversion have confirmed that mtDNA methylation, while sparser than in nuclear DNA, is enriched in regulatory and tRNA-coding regions and varies across tissues, developmental stages, and physiological states [203,213,214]. Emerging work further demonstrates that mitochondrial cytosine modifications are metabolically responsive, fluctuating with intermediates of the TCA cycle, NAD^+^/NADH ratios, and ROS levels, thereby coupling mitochondrial metabolism to its own epigenome [215,216,217]. Mitochondrial 5mC and 5hmC modifications are now recognized to influence replication-origin activity, transcriptional asymmetry, and gene expression plasticity within the organelle. Collectively, these studies establish mtDNA as a metabolically sensitive epigenetic platform, where cytosine methylation and hydroxymethylation act as dynamic modulators of mitochondria-nucleus communication, oxidative-stress adaptation, and disease susceptibility.

### 3.2. MOF/KAT8

Recent studies highlight that the histone acetyltransferase MOF (males absent on the first, KAT8)—classically recognized for catalyzing histone H4 lysine 16 acetylation (H4K16ac) within the nuclear genome—also performs essential regulatory functions in mitochondria, revealing its dual localization and multifaceted roles in coordinating gene expression and metabolism. Early investigations demonstrated that MOF/KAT8 forms a mitochondrial complex with KAT8 Regulatory Non-Specific Lethal complex (KANSLs) subunits, where it promotes mitochondrial transcription and respiratory-chain activity through acetylation of the mitochondrial transcription factor TFAM [218]. This modification enhances TFAM’s DNA-binding ability and stability, thereby facilitating efficient mitochondrial gene expression and oxidative phosphorylation.

Subsequent research expanded this paradigm by identifying additional mitochondrial substrates of MOF/KAT8. For instance, COX17, a copper chaperone critical for cytochrome c oxidase assembly, is acetylated by MOF, an event that preserves mitochondrial integrity and supports respiratory-chain function [219,220]. Likewise, MOF-mediated acetylation of ATP5β, a core subunit of ATP synthase, was recently shown to sustain mitochondrial respiration and protect against pressure-overload-induced cardiac energy failure [221].

Collectively, the MOF/KAT8–KANSL complex represents a molecular bridge between nuclear epigenetic control and metabolic regulation. Its acetyltransferase activity not only maintains mitochondrial transcriptional competence but also orchestrates bioenergetic adaptation and metabolic homeostasis, underscoring MOF’s dual identity as both a nuclear epigenetic enzyme and a mitochondrial metabolic regulator.

### 3.3. GCN5L

The mitochondrial homolog of the nuclear histone acetyltransferase GCN5, termed GCN5-like protein 1 (GCN5L1), has emerged as a pivotal regulator of mitochondrial protein acetylation and metabolic signaling. Unlike its nuclear counterpart, which modifies histones to regulate chromatin structure and gene expression, GCN5L1 localizes to the mitochondrial matrix and functions as a non-histone lysine acetyltransferase, directly modifying metabolic enzymes and structural proteins critical for mitochondrial function [222,223].

Within mitochondria, GCN5L1 acetylates diverse enzymes involved in oxidative metabolism—including hydroxyacyl-CoA dehydrogenase trifunctional multienzyme complex subunit alpha (HADHA), NADH-ubiquinone oxidoreductase subunit B8 (NDUFB8), and superoxide dismutase 2, mitochondrial (SOD2; MnSOD)—thereby influencing energy production, redox balance, and lipid utilization. Together with its antagonistic deacetylase SIRT3 (see below), GCN5L1 forms a reversible acetylation-deacetylation circuit that tunes mitochondrial metabolism in response to nutrient availability and oxidative stress. Functionally, GCN5L1 serves as a metabolic rheostat that regulates fatty acid oxidation in a tissue-specific manner. In the liver, GCN5L1 acetylates and inhibits β-oxidation enzymes, acting as a metabolic “brake” under nutrient-rich conditions, whereas its downregulation promotes fatty acid oxidation and ATP production during fasting or caloric restriction [224]. In cardiomyocytes, by contrast, GCN5L1-mediated acetylation correlates with enhanced fatty acid oxidation capacity and improved oxidative performance [225]. In addition, GCN5L1 regulates mitophagy—the selective degradation of mitochondria—by acetylating core mitophagy regulators and mitochondrial dynamics proteins, thereby preserving mitochondrial integrity and energy efficiency [226].

Recent evidence extends the role of GCN5L1 beyond metabolism to mitochondrial dynamics and stress adaptation. In stress and disease contexts, GCN5L1 influences mitochondrial fission and bioenergetic resilience. Specifically, GCN5L1 acetylates Drp1 downstream of the CDK5–AMPK signaling axis, promoting mitochondrial fragmentation and neuronal injury during cerebral ischemia [227]. These findings highlight GCN5L1 as both a sensor and effector of mitochondrial stress, linking nutrient status and bioenergetic demand to structural remodeling of mitochondria.

Taken together, mitochondrial GCN5L1 integrates nutrient availability, redox equilibrium, and mitochondrial dynamics. Through its acetylation of TCA cycle enzymes, respiratory complexes, and mitochondrial fission/fusion regulators, GCN5L1 safeguards the balance between energy production, lipid oxidation, and mitochondrial quality management—core processes that underpin metabolic flexibility and survival under physiological and stress conditions.

### 3.4. Sirtuins (SIRTs)

The sirtuin family (SIRT1-SIRT7) comprises highly conserved deacylases that modulate chromatin structure, metabolism, and stress adaptation. Originally characterized as nuclear transcriptional silences linked to lifespan extension, sirtuins are now recognized as central regulators of mitochondrial bioenergetics, redox balance, and mitochondria-nuclear communication [228,229].

Among them, SIRT3, SIRT4, and SIRT5 reside predominantly in the mitochondrial matrix, where they orchestrate metabolic fluxes and oxidative stress responses. SIRT3 acts as the principal mitochondrial deacetylase, activating multiple enzymes including IDH2, SDHA, and MnSOD, thereby enhancing ATP production and antioxidant defense [230,231]. Loss of SIRT3 results in hyperacetylation of mitochondrial proteins, impaired electron transport chain activity, and increased ROS, linking its deficiency to metabolic syndrome, cardiac hypertrophy, and age-related dysfunction. SIRT4 and SIRT5 fine-tune mitochondrial metabolism through non-deacetylase activities. SIRT4 exhibits ADP-ribosyltransferase and lipoamidase activities, thereby modulating amino acid catabolism and insulin secretion, whereas SIRT5 functions as a desuccinylase, demalonylase, and deglutarylase, regulating oxidative metabolism and the detoxification of reactive intermediates [232]. Together, these mitochondrial sirtuins preserve metabolic flexibility and redox equilibrium, enabling cells to adapt to nutrient fluctuations and oxidative challenges, thereby promoting cellular resilience and longevity.

Beyond the mitochondria, nuclear sirtuins—SIRT1, SIRT6, and SIRT7—also exert profound control over mitochondrial physiology, reinforcing the concept of bidirectional nucleus-mitochondria communication. SIRT1 stimulates mitochondrial biogenesis by deacetylating PGC-1α, NRF1, and FOXO3, thereby upregulating nuclear-encoded mitochondrial genes [229]. SIRT6 and SIRT7, while primarily nuclear, modulate mitochondrial homeostasis indirectly via transcription, mitochondrial unfolded protein (UPR^mt) signaling, and maintenance of proteostasis [233,234]. Taken together, nuclear and mitochondrial sirtuins constitute a spatially distributed NAD^+^-dependent network that coordinates chromatin regulation with mitochondrial function within the broader metabolic–epigenetic axis described above [228,232,235].

### 3.5. HDAC3

Although traditionally recognized as a nuclear class I histone deacetylase that mediates chromatin remodeling and transcriptional repression [236], HDAC3 also exerts non-canonical mitochondrial functions that extend its regulatory influence into energy metabolism and inflammation. In macrophages and hepatocytes, HDAC3 translocates to mitochondria, where it associates physically with the ATP synthase complex. Within mitochondria, HDAC3 deacetylates the HADHA at K303, thereby fine-tuning fatty-acid oxidation and mitochondrial adaptation. This activity shapes the threshold of NLRP3 (NOD-like receptor family pyrin domain containing 3) inflammasome activation and IL-1β secretion, ultimately modulating macrophage polarization and inflammatory responses [237,238]. Collectively, mitochondrial HDAC3 functions as a homeostatic regulator that integrates acetylation control of metabolic enzymes with immune signaling. By coupling mitochondrial deacetylation to fatty acid oxidation efficiency and ATP synthase function, HDAC3 links cellular metabolic state to innate immune tone. Yet, the mechanisms underlying its mitochondrial import, substrate selectivity, and context-specific regulation remain incompletely understood—representing a promising frontier for future investigation at the interface of mitochondrial epigenetics and immunometabolism.

### 3.6. Histones

For decades, mtDNA packaging was attributed primarily to TFAM (mitochondrial transcription factor A), a high-mobility group protein essential for mtDNA stability, replication, and transcription [239,240]. However, accumulating evidence has begun to challenge this long-held view, suggesting that canonical histones may also participate in the structural and regulatory organization of mtDNA [241,242,243].

Proteomic and biochemical investigations have detected core histones within mitochondria across multiple species. Early biochemical studies identified histones H2A/H2B in mitochondria outer membrane or membrane-associated fractions [244]. DNase I footprinting and atomic force microscopy later revealed nucleosome-like periodic protection patterns across the mtDNA, reminiscent of chromatinized DNA rather than TFAM-only coverage [241]. These observations suggest a more complex mtDNA organization that parallels features of nuclear chromatin. Further cross-species analyses reinforced the presence of histones within mitochondria. In trypanosomatid mitochondria, Kapeller et al. (2011) demonstrated mitochondrial localization of linker histone H1 [245]. In plants, Zanin et al. [246] reported the presence of histone H3 within mitochondria, while in *C. elegans*, Sural et al. showed that histone H4 localizes to mitochondria, mediated by the Heat Shock Factor Binding protein 1/Heat Shock Factor 1 signaling axis (HSB-1/HSF-1 signaling axis), directly modulating mtDNA transcription and organismal lifespan [243]. Together, these studies reveal that mitochondrial histone dynamics are conserved across evolution and can influence both mitochondrial gene expression and organism physiology.

More recently, Shi et al. [247] provided direct evidence that ubiquitinated histone H2A (H2Aub)—traditionally regarded as a nuclear Polycomb repressive complex 1 (PRC1)-dependent modification mark—also occurs within mitochondria. Biochemical fractionation of affinity-purified mitochondria and proximal ligation assays confirm this modification in mitochondrial fractions, while knockout of PRC1 ubiquitin ligase subunits Ring1/RNF2 abolished the signal. This unexpected discovery suggests that H2Aub participates in mtDNA packaging, transcriptional repression, and chromatin-like remodeling, revealing a previously unrecognized layer of mitochondrial epigenetic regulation.

Collectively, these findings indicate that canonical histones and their modifications may localize within mitochondria, contributing to mtDNA compaction, transcriptional regulation, and mitochondria genome stability. This emerging view challenges the traditional model of TFAM-exclusive nucleoid organization and supports a mitochondrial chromatin architecture that integrates nuclear histone dynamics with mitochondrial gene expression, thereby linking epigenetic regulation to bioenergetics, redox signaling, and longevity.

### 3.7. PRC1/USP16

Recent research has unveiled an unexpected mitochondrial role for canonical chromatin regulator Polycomb Repressive Complex 1 (PRC1) and the deubiquitinase USP16. Traditionally known for opposing functions in the regulation of histone H2A ubiquitination (H2Aub) within the nucleus, significant fractions of these enzymes have now been shown to localize to mitochondria, where they execute crucial roles in maintaining organelle integrity and bioenergetic homeostasis. Using biochemical fractionation, confocal microscopy, and proximity ligation assays, Shi et al. [247] demonstrated that core PRC1 components (RING1, RING2/RNF2) and USP16 co-localize with mitochondrial markers such as TOM20 and HSP60, and are resistant to proteinase K digestion—supporting a mitochondrial matrix localization. Additionally, co-immunoprecipitation assays further revealed that PRC1 and USP16 physically interact with mitochondrial ubiquitin and matrix enzymes, such as DBT, confirming their functional presence within the organelle rather than contamination from nuclear fractions.

Functionally, PRC1–USP16 activity is essential for mitochondrial morphology, respiration, and proteome stability. Depletion of RNF2 (RING2) or Usp16 by siRNA, gene knockout, or targeted degradation from the mitochondria results in reduced oxygen consumption rate (OCR), loss of mitochondrial membrane potential, and impaired integrity. Proteomic analyses revealed that PRC1 loss causes downregulation of OXPHOS and TCA cycle components, accompanied by increased mitochondrial stress signaling [247]. These defects are accompanied by ubiquitin pathway disruptions, including altered mitochondrial protein ubiquitination patterns resembling H2Aub-like 23 kDa bands, indicating that histone-like ubiquitination events may occur within mitochondria. Such findings suggest that PRC1 and USP16 form a mitochondrial ubiquitination–deubiquitination axis that preserves protein quality control and respiratory efficiency.

The identification of H2Aub-like species in purified mitochondria raises intriguing parallels between mitochondrial nucleoids and nuclear chromatin. Combined with earlier reports identifying core histones (H1, H3, H4) and nucleoid-associated proteins within mitochondria, these findings point toward a chromatin-like layer of regulation acting on mtDNA. PRC1- and USP16-mediated modification of nucleoid-associated proteins may influence mtDNA compaction, transcriptional accessibility, or replication dynamics, extending the concept of epigenetic regulation beyond the nucleus. This hypothesis aligns with DNase-footprinting evidence indicating conserved protein–DNA footprints on mtDNA, similar to those on nuclear DNA [241]. Collectively, these observations suggest a broader framework for ubiquitin signaling and chromatin-like regulation within mitochondria.

Given that PRC1 and USP16 are central to developmental gene regulation and cellular aging, their mitochondrial localization provides a mechanistic bridge linking nuclear transcriptional repression to mitochondrial energetics. Dysregulation of this axis could contribute to metabolic reprogramming, oxidative stress susceptibility, and mitochondrial dysfunction in diseases such as cancer, neurodegeneration, and premature aging. Thus, mitochondrial PRC1 and USP16 exemplify how canonical nuclear regulators are repurposed to sustain organelle homeostasis and cellular metabolic balance.

The discovery of mitochondrial-localized complexes such as PRC1, USP16, and MOF/KAT8, together with evidence for mitochondrial H2Aub, introduces a new layer of organelle-specific epigenetic control. Taken together, these observations extend our view of nuclear–mitochondrial communication, demonstrating that canonical epigenetic regulators and histone modifications not only on nuclear chromatin but also directly within mitochondria to influence genome organization, metabolism, and stress resilience, although they still require independent replication and mechanistic dissection. This emerging dual regulatory framework—integrating nuclear and mitochondrial epigenetic mechanisms—illuminates how chromatin dysregulation may drive mitochondrial dysfunction in aging and disease, and highlights new opportunities to restore mitochondrial health via epigenetic modulation.

## 4. Bidirectional Mitochondria–Nucleus Communication

**Figure 4:**
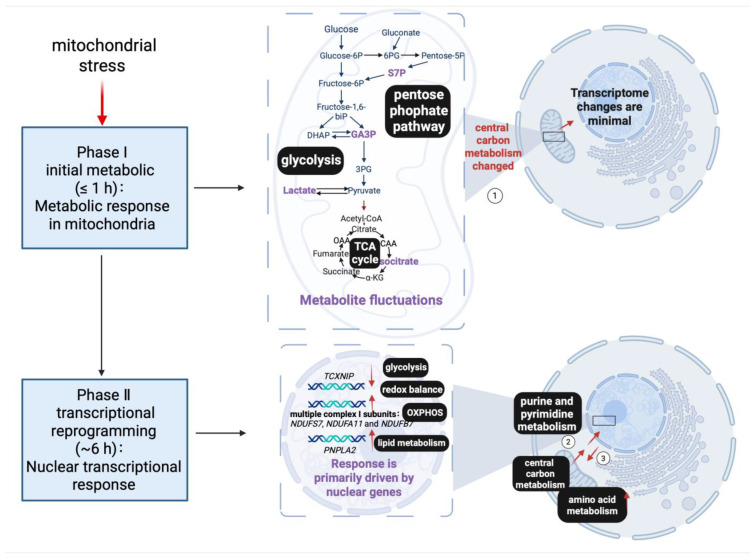
Biphasic cellular response to mitochondrial stress: an early metabolic adaptation followed by a delayed nuclear transcriptional reprogramming. Mitochondrial stress first elicits a rapid Phase I response (≤1 h), characterized by pronounced fluctuations in central carbon metabolism within mitochondria, including glycolysis, the TCA cycle, and the pentose phosphate pathways, while nuclear transcriptome changes remain minimal (1). Phase II (~6 h) features sustained perturbation of central carbon metabolism and expansion to additional pathways, including purine and pyrimidine metabolism (2). At this time point, amino acid metabolism shows a striking shift, with most amino acid–related pathways strongly upregulated (3). Along with these metabolic shifts, a broad nuclear transcriptional response emerges by ~6 h: genes controlling glycolysis, oxidative phosphorylation, redox balance, and lipid metabolism (e.g., *TXNIP*, *PNPLA2*, and genes encoding multiple respiratory complex I subunits such as *NDUFS7*) are differentially expressed. Thus, mitochondrial stress responses are first encoded as metabolic shifts. Persistent stress leads to modulations of nuclear gene-expression programs. Created in BioRender. Xu, Y. (2026) https://BioRender.com/tnvanfb (accessed on 27 November 2025).

As summarized above, apart from the established roles in bioenergetics and biosynthesis, mitochondria are signaling organelles that communicate their fitness to the nucleus, triggering transcriptional programs to adapt homeostasis to stress, essential for organismal health and aging. Notably, mitochondrial perturbations trigger an exceptionally rapid and coordinated nuclear response. Integrated transcriptomic and metabolomic analyses reveal a biphasic adaptive program: an initial metabolic phase followed by a delayed but robust transcriptional reprogramming [238]. Within the first hour, changes observed at the transcriptomic level are minimal, yet metabolites associated with the TCA cycle, glycolysis, and pentose phosphate pathway fluctuate sharply—most notably isocitrate, which consistently marks mitochondrial perturbation (Figure 4).

Among tested stressors, antimycin A, a mitochondrial ETC inhibitor produced by *Streptomyces*, causes the strongest metabolic disruption, altering over 50 metabolites within one hour. By 6 h, these changes in central carbon metabolism persisted, while additional pathways, including purine and pyrimidine metabolism, also became frequently perturbed. In contrast, amino acid metabolism displayed a striking pattern at 6 h, with most amino acid–related pathways strongly upregulated. Along with these metabolic alternations, a broad nuclear transcriptional response emerges by 6 h, encompassing hundreds of differentially expressed genes (DEGs), with ~371 conserved across 4 stressors [238]. These DEGs cluster in pathways linked to glycolysis, OXPHOS, lipid metabolism, and redox balance, including early responders such as *TXNIP*, *PNPLA2*, and *NDUFS7*. Notably, the later phase of adaptation (∼6 h) was dominated by nuclear gene regulation, with no comparable early changes reported for mtDNA-encoded transcripts, indicating that acute adaptation is primarily orchestrated through nuclear gene regulation rather than direct mitochondrial transcription.

These insights highlight how mitochondrial stress initially manifests as quick changes in metabolic and redox states. These changes then trigger a delayed but widespread reprogramming of nuclear transcription. This process, driven by metabolites, creates a two-way communication loop between mitochondria and the nucleus, connecting mitochondrial health with the state of chromatin and overall cell stability.

## 5. Methodological Innovation: Mitochondria-Targeted AID System

**Figure 5:**
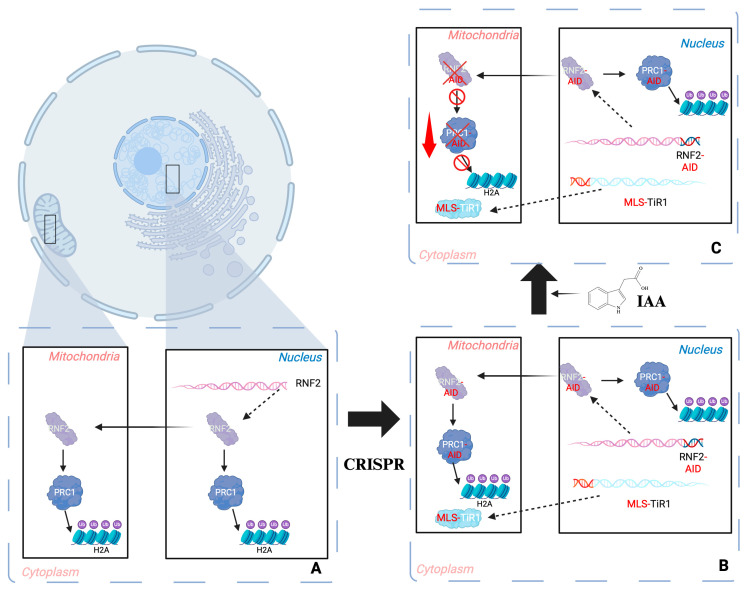
Mitochondria-targeted AID system enables compartment-specific degradation of PRC1/RNF2. (**A**). Under basal conditions, the ubiquitin ligase RNF2-containing PRC1 complexes encoded in the nucleus are present in both mitochondria and the nucleus. (**B**). CRISPR-mediated genome editing introduces an auxin-inducible degron (AID) tag into the endogenous RNF2 gene. Meanwhile, a transgene in the nucleus expresses a mitochondria-localized auxin receptor TIR1 (MLS-TIR1), which promotes RNF2-AID in PRC1 in mitochondria to be specifically recognized. (**C**). Upon addition of the indole-3-acetic acid (IAA) inducer, mitochondrial RNF2 AID in PRC1 complex is selectively degraded by the mitochondria-targeted TIR1 machinery, while nuclear RNF2-AID in PRCI1 remains intact. This approach enables spatially restricted depletion of mitochondrial PRC1 to dissect its organelle-specific functions without globally abolishing nuclear PRC1 activity. Some nucleosomes are shown to be ubiquitinated. Abbreviations: AID, auxin-inducible degron; PRC1, Polycomb repressive complex 1; RNF2, RING finger protein 2; MLS, mitochondrial localization sequence; TIR1, Auxin receptor transport inhibitor response 1; IAA, indole-3-acetic acid. Created in BioRender. Xu, Y. (2026) https://BioRender.com/tnvanfb (accessed on 27 November 2025).

There are experimental limitations to studying regulatory proteins that are dually localized to the nucleus and mitochondria. Conventional knockdown or knockout methods deplete both pools simultaneously, confounding interpretation of whether observed phenotypes originate from nuclear or mitochondrial activity. To overcome this limitation, a mitochondria-targeted auxin-inducible degron (AID) system, enabling acute, reversible, and organelle-specific degradation of proteins, was developed [237]. This tool provides unmatched temporal precision and subcellular specificity. Its application to RNF2 (RING2), the H2Aub catalytic subunit of PRC1, demonstrated that targeted mitochondrial degradation of RNF2 markedly impairs mitochondrial respiration, directly linking its mitochondrial pool to bioenergetic maintenance (Figure 5).

This proof-of-concept study validates the efficacy of the mitochondria-targeted AID system as a powerful and versatile instrument for dissecting the organelle-specific roles of multifunctional proteins. More broadly, it enables systematic exploration of how epigenetic regulators, traditionally viewed as nuclear factors, operate with mitochondria to influence metabolic adaptation, genome organization, and cellular signaling. By separating mitochondria from nuclear contributions, this approach opens new frontiers for uncovering previously hidden dimensions of nuclear-mitochondrial communications, with far-reaching implications for understanding energy metabolism, stress response, and aging.

## 6. Perspective

The discoveries summarized in this review emphasize an emerging view: mitochondria are not just metabolic powerhouses but integral players in shaping the epigenetic landscape of the cell. By supplying essential metabolites such as ATP, acetyl-CoA, NAD^+^, α-ketoglutarate, and other metabolites, mitochondria directly influence the activity of chromatin-modifying enzymes and nuclear transcriptional programs. This metabolic-epigenetic interface ensures that cellular gene expression programs are tightly coupled to cellular energy states.

Importantly, the detection of classical epigenetic regulators within mitochondria reveals that this communication is bidirectional. Nuclear-mitochondria crosstalk therefore emerges as a reciprocal regulatory circuit: while mitochondrial metabolic signaling influences nuclear chromatin and transcription, certain nuclear epigenetic factors in turn operate within mitochondria to regulate the production of ATP and metabolites. Together, these insights support an integrated view in which mitochondria serve as a central and versatile regulator of epigenetic state, with implications spanning metabolic adaptation, stress resilience, and organismal aging.

The development of advanced experimental platforms, notably the mitochondria-targeted AID system, offers a strong approach to interrogate this bidirectional communication with unprecedented spatial and temporal precision. By enabling the acute and selective depletion of mitochondrial proteins, the mitochondria-AID strategy overcomes the limitations of conventional knockouts and RNA interference (RNAi) methods, allowing the establishment of causal relationships between mitochondrial perturbations and nuclear epigenetic remodeling. The successful implementation of a mitochondria-AID system in cell-based studies opens an exciting frontier for in vivo applications. Extending this approach to transgenic mouse models expressing AID-tagged versions of mitochondrial proteins would enable tissue-specific and temporally controlled protein degradation. Applying this system in vivo would thus allow direct mechanistic dissection of how mitochondrial epigenetic factors, including PRC1, USP16, and many histone-modifying enzymes, govern developmental processes, tissue homeostasis, aging, and disease progression. Moreover, coupling this system with multi-omics analyses, including transcriptomics, proteomics, epigenomics, and metabolomics, could yield a dynamic, system-level understanding of how mitochondrial perturbations reshape nuclear gene expression, chromatin states, and cellular metabolism in real time. Together, these methodological advances promise to transform our understanding of mitochondrial contributions to epigenetic regulation, bridging molecular insights with physiological outcomes.

The dual regulatory framework integrating nuclear and mitochondrial epigenetic mechanisms helps explain how chromatin dysregulation may contribute to mitochondrial dysfunction in aging and disease, while also highlighting emerging opportunities to restore mitochondrial health through epigenetic modulation.

## Data Availability

No new data were created or analyzed in this study.
