# Correction: Xu et al. Mitochondria and Epigenetic Regulation: Bidirectional Crosstalk and Emerging Mitochondria-Targeted Degron Tools. *Cells* 2026, *15*, 95

**DOI:** 10.3390/cells15060530

**Published:** 2026-03-17

**Authors:** Yingwei Xu, Xiaokun Jian, Lei Shi, Lisa S. Shock, Lanming Chen, Louise T. Chow, Hengbin Wang

**Affiliations:** 1 Department of Internal Medicine, Division of Hematology, Oncology and Palliative Care, Massey Comprehensive Cancer Center, Virginia Commonwealth University, Richmond, VA 23298, USA; yingweixu2023@gmail.com (Y.X.); doudou246@163.com (L.S.); lisa.shock@vcuhealth.org (L.S.S.); 2 College of Food Science and Technology, Shanghai Ocean University, Shanghai 201306, China; lmchen@shou.edu.cn; 3 School of Basic Medical Sciences, Xinxiang Medical University, Xinxiang 453003, China; 4 Department of Microbiology and Immunology, School of Medicine, Virginia Commonwealth University, Richmond, VA 23284, USA; 5 Department of Biochemistry and Molecular Genetics, University of Alabama at Birmingham, Birmingham, AL 35294, USA; ltchow@uab.edu

In the original publication [1], there was an error in the attribution of experimental findings and in the corresponding reference numbering in Section 2.3. The first sentence, describing long-term innate immune memory, incorrectly attributed the results of Ziogas et al. (2025) to Yuan et al., and the cited reference number did not correspond to the correct article. With this correction, the new reference for Ziogas et al. [2] has been inserted as Reference 75, and the former Reference 75 (Yuan et al. [3]) and all subsequent references have been renumbered accordingly. A correction has been made to Section 2.3, “Histone Lactylation”, paragraph 2:

“In innate immunity, Ziogas et al. [75] showed that lactate-driven H3K18la persists in monocytes for at least 90 days after BCG vaccination, serving as a long-lived epigenetic mark of trained innate immune memory. Lactate-induced H3K18la also promotes macrophage M1 polarization and vascular inflammation in models of abdominal aortic aneurysm, illustrating how Kla connects local metabolic changes to chronic inflammatory disease [76].”

The authors state that the scientific conclusions are unaffected. This correction was approved by the Academic Editor. The original publication has also been updated.
